# Supplementary material for: Auditory mismatch responses are differentially sensitive to changes in muscarinic acetylcholine versus dopamine receptor function
Source: eLife. 2022 May 3;11:e74835. doi: 10.7554/eLife.74835 (PMC9098218; doi:10.7554/eLife.74835)
Supplement: Supplementary file 1. — Tables show the results for the main contrasts reported in the main text when excluding data sets due to lack of behavioral data or low performance in the visual distraction task. [file elife-74835-supp1.docx]

## Supplementary File 1

## Control analyses: Excluding data sets based on the distraction task

| **Study 1:** $N=67$ | cluster | $x[\mathrm{mm}]$ | $y[\mathrm{mm}]$ | $z[\mathrm{ms}]$ | $t_{62}$ | $Z_{\equiv}$ | $p_{FWE}$ | $k_{E}$ | $tw_{sig}$ [ms] |
| --- | --- | --- | --- | --- | --- | --- | --- | --- | --- |
| **A** standards > deviants | 1 | -13 | 2 | 172 | 15.73 | Inf | 0.000 | 8060 | 100 - 228 |
|  |  | 8 | -3 | 180 | 15.26 | Inf | 0.000 |  |  |
|  | 2 | 4 | 2 | 400 | 9.17 | 7.26 | 0.000 | 1181 | 364 - 400 |
|  | 3 | -8 | 50 | 276 | 6.80 | 5.85 | 0.000 | 264 | 240 - 300 |
|  |  | -4 | 45 | 248 | 5.03 | 4.59 | 0.007 |  |  |
|  | 4 | 0 | -95 | 304 | 5.13 | 4.66 | 0.005 | 92 | 288 - 332 |
|  | 5 | -60 | -57 | 268 | 4.83 | 4.44 | 0.013 | 191 | 240 - 284 |
|  |  | -60 | -9 | 256 | 4.75 | 4.37 | 0.016 |  |  |
|  |  | -60 | -36 | 260 | 4.74 | 4.37 | 0.017 |  |  |
| **B** deviants > standards | 1 | 17 | 72 | 156 | 13.99 | Inf | 0.000 | 1524 | 100 - 236 |
|  |  | 4 | 72 | 212 | 9.74 | 7.56 | 0.000 |  |  |
|  |  | -30 | 61 | 184 | 7.67 | 6.41 | 0.000 |  |  |
|  | 2 | -47 | -68 | 180 | 13.96 | Inf | 0.000 | 5986 | 100 - 328 |
|  |  | 64 | -62 | 200 | 13.52 | Inf | 0.000 |  |  |
|  |  | 42 | -78 | 168 | 11.49 | Inf | 0.000 |  |  |
|  | 3 | -51 | -30 | 400 | 6.18 | 5.44 | 0.000 | 340 | 372 - 400 |
|  | 4 | 34 | -52 | 364 | 6.12 | 5.39 | 0.000 | 418 | 352 - 400 |
|  |  | 47 | -52 | 400 | 5.60 | 5.02 | 0.001 |  |  |
|  | 5 | 64 | -62 | 100 | 4.87 | 4.46 | 0.012 | 9 | 100 - 112 |
|  | 6 | 0 | 72 | 400 | 4.67 | 4.31 | 0.021 | 8 | 400 - 400 |
|  |  | -17 | 72 | 400 | 4.49 | 4.17 | 0.035 |  |  |
| **C** mismatch: AMI > BIP | 1 | 13 | 67 | 160 | 5.26 | 4.76 | 0.003 | 70 | 148 - 168 |
| **D** stable MMN > volatile MMN | 1 | 17 | -19 | 204 | 5.25 | 4.75 | 0.004 | 407 | 180 - 216 |
|  |  | -17 | -25 | 196 | 5.20 | 4.72 | 0.005 |  |  |
|  | 2 | -8 | 45 | 256 | 4.70 | 4.33 | 0.022 | 19 | 252 - 260 |
| **E** volatile MMN > stable MMN | 1 | 42 | -78 | 196 | 5.12 | 4.66 | 0.006 | 69 | 188 - 212 |
|  |  | 30 | -89 | 204 | 4.89 | 4.48 | 0.013 |  |  |
|  |  | 55 | -68 | 196 | 4.83 | 4.43 | 0.015 |  |  |
|  | 2 | -60 | -57 | 208 | 5.00 | 4.56 | 0.009 | 15 | 200 - 216 |
|  | 3 | 4 | 72 | 212 | 4.54 | 4.20 | 0.036 | 7 | 208 - 212 |
|  | 4 | 13 | -95 | 208 | 4.49 | 4.16 | 0.041 | 2 | 208 - 212 |
| **F** stable > volatile | 1 | 4 | -62 | 284 | 5.54 | 4.98 | 0.001 | 156 | 272 - 296 |
|  | 2 | 4 | -95 | 144 | 4.88 | 4.47 | 0.013 | 35 | 140 - 152 |
|  | 3 | 0 | 61 | 148 | 4.50 | 4.17 | 0.039 | 2 | 144 - 148 |

**Table S1** Significant activations for a reduced sample size (*N*=67) in study 1. Table shows whole-volume corrected significant effects after excluding three data sets due to lack of behavioral data and one dataset based on low performance in the distraction task (hit rate<75%). The results reported for the full sample (*N*=71) in Table 1, Table 4 and Appendix 2—table 1 hold.

**Table S2** Significant activations for a reduced sample size (*N*=77) in study 2. Table shows whole-volume corrected significant effects after excluding one data set based on low performance in the distraction task (hit rate<75%). All results reported for the full sample (*N*=78) in Table 2 and Appendix 2—table 1 hold, except for the pharmacological effect (GAL > PLA) on stability ERPs reported in Appendix 2—table 1.

| **Study 2:** $N=77$ | cluster | $x[\mathrm{mm}]$ | $y[\mathrm{mm}]$ | $z[\mathrm{ms}]$ | $t_{72}$ | $Z_{\equiv}$ | $p_{FWE}$ | $k_{E}$ | $tw_{sig}$ [ms] |
| --- | --- | --- | --- | --- | --- | --- | --- | --- | --- |
| **A** standards > deviants | 1 | 13 | -9 | 176 | 13.99 | Inf | 0.000 | 7462 | 100 - 216 |
|  |  | 4 | 18 | 160 | 3.79 | Inf | 0.000 |  |  |
|  |  | 42 | -19 | 124 | 10.61 | Inf | 0.000 |  |  |
|  | 2 | 0 | -3 | 396 | 8.36 | 6.95 | 0.000 | 1317 | 364 - 400 |
|  | 3 | 4 | -95 | 296 | 7.25 | 6.25 | 0.000 | 688 | 244 - 332 |
|  |  | 30 | -89 | 280 | 6.64 | 5.83 | 0.000 |  |  |
|  |  | 51 | -68 | 256 | 5.53 | 5.02 | 0.001 |  |  |
|  | 4 | 4 | 61 | 288 | 6.07 | 5.43 | 0.000 | 290 | 256 - 304 |
|  |  | 0 | 56 | 268 | 5.92 | 5.32 | 0.000 |  |  |
|  | 5 | -47 | -68 | 256 | 5.78 | 5.21 | 0.000 | 298 | 232 - 284 |
|  |  | -60 | -57 | 260 | 5.61 | 5.09 | 0.001 |  |  |
| **B** deviants > standards | 1 | -42 | -73 | 172 | 14.11 | Inf | 0.000 | 5568 | 100 - 216 |
|  |  | 55 | -68 | 196 | 10.81 | Inf | 0.000 |  |  |
|  |  | -38 | -73 | 124 | 10.15 | Inf | 0.000 |  |  |
|  | 2 | -13 | -30 | 256 | 7.31 | 6.29 | 0.000 | 2724 | 232 - 328 |
|  |  | -26 | -14 | 288 | 6.85 | 5.98 | 0.000 |  |  |
|  |  | 8 | -9 | 304 | 6.61 | 5.81 | 0.000 |  |  |
|  | 3 | 38 | -68 | 400 | 6.46 | 5.71 | 0.000 | 273 | 368 - 400 |
|  | 4 | 4 | 72 | 388 | 5.86 | 5.28 | 0.000 | 146 | 368 - 400 |
|  | 5 | 68 | 18 | 196 | 5.39 | 4.92 | 0.002 | 16 | 172 - 204 |
|  | 6 | -60 | -9 | 400 | 4.95 | 4.57 | 0.008 | 36 | 396 - 400 |
|  | 7 | -34 | -62 | 396 | 4.86 | 4.50 | 0.010 | 50 | 388 - 400 |
| **C** volatile > stable | 1 | 4 | 18 | 384 | 4.63 | 4.32 | 0.025 | 32 | 380 - 388 |
|  | 2 | 0 | 40 | 268 | 4.47 | 4.18 | 0.041 | 3 | 268 - 272 |
|  | 3 | -21 | -19 | 356 | 4.41 | 4.13 | 0.050 | 1 | 356 - 356 |
| **D** stability: PLA > LEV | 1 | -38 | -14 | 264 | 4.70 | 4.37 | 0.020 | 9 | 260 - 264 |
